# Nanopore MinION Sequencing Generates a White Spot Syndrome Virus Genome from a Pooled Cloacal Swab Sample of Domestic Chickens in South Africa

**DOI:** 10.3390/microorganisms11112802

**Published:** 2023-11-18

**Authors:** Ravendra P. Chauhan, Ronen Fogel, Janice Limson

**Affiliations:** Biotechnology Innovation Centre, Rhodes University, Makhanda 6139, Eastern Cape, South Africa; ravendrachauhan@hotmail.com (R.P.C.); r.fogel@ru.ac.za (R.F.)

**Keywords:** domestic chickens, faecal virome, metagenomic sequencing, Nanopore MinION sequencing, SISPA, White spot syndrome virus.

## Abstract

White spot syndrome virus is a highly contagious pathogen affecting shrimp farming worldwide. The host range of this virus is primarily limited to crustaceans, such as shrimps, crabs, prawns, crayfish, and lobsters; however, several species of non-crustaceans, including aquatic insects, piscivorous birds, and molluscs may serve as the vectors for ecological dissemination. The present study was aimed at studying the faecal virome of domestic chickens (*Gallus gallus domesticus*) in Makhanda, Eastern Cape, South Africa. The cloacal swab specimens (*n* = 35) were collected from domestic chickens in December 2022. The cloacal swab specimens were pooled—each pool containing five cloacal swabs—for metagenomic analysis using a sequence-independent single-primer amplification protocol, followed by Nanopore MinION sequencing. While the metagenomic sequencing generated several contigs aligning with reference genomes of animal viruses, one striking observation was the presence of a White spot syndrome virus genome in one pool of cloacal swab specimens. The generated White spot syndrome virus genome was 273,795 bp in size with 88.5% genome coverage and shared 99.94% nucleotide sequence identity with a reference genome reported in China during 2018 (GenBank accession: NC_003225.3). The Neighbour-Joining tree grouped South African White spot syndrome virus genome with other White spot syndrome virus genomes reported from South East Asia. To our knowledge, this is the first report of a White spot syndrome virus genome generated from domestic chickens. The significance of White spot syndrome virus infection in domestic chickens is yet to be determined.

## 1. Introduction

White spot syndrome virus (WSSV; family *Nimaviridae*)—the sole member of the genus Whispovirus—is an enveloped, double-stranded DNA virus with a circular genome [1]. WSSV is of high significance for the global shrimp industry and causes devastating white spot disease (WSD) in farmed shrimps [2,3,4]. During WSD, the WSSV causes clinical signs of white spots ranging from 0.5 to 3.0 mm in diameter, present on the appendages, exoskeletons, and inside the epidermises of infected shrimps [5,6]. Some of the other clinical signs of WSSV infection in shrimps may include a sharp reduction in food intake, lethargy, looseness of cuticle, and red discoloration of body and appendages [6]. An experimental study using kuruma shrimp (*Marsupenaeus japonicas*) suggested that WSSV may replicate within cells of antennal glands, the epithelial cells in gills and the foregut, causing dysfunction of these organs, subsequently leading to death [7].

WSD is a highly virulent disease affecting cultured shrimp, typical losses range between 80 and 100% mortality within 3–10 days of outbreak [8,9]. The first reports of WSD occurred in Taiwan in 1992 [10], followed by Japan [11] and China during 1993 [3], where the disease caused high mortality in cultured shrimps, exceeding 80% within a week after the outbreak [3,10,11]. Since then, WSD has caused disease outbreaks in various crustacean species in many countries in Asia [1,3,12,13,14,15], Europe [16], Australia [17], and the Americas [18,19,20]. Apart from shrimp, WSD has also been reported in other commercially important crustaceans, including prawns [17], crabs [15], crayfish [21], and lobsters [22,23].

As an estimate, during 2009–2018, the Asian shrimp industry experienced an annual loss of approximately 4 billion USD [24]. Given the global market value of shrimp farming, which was estimated to be 38.4 billion USD in 2018 [25], the risk of inflicting significant mortality makes the detection and monitoring of WSSV important.

The ecological dispersal and retention of WSSV is thought to be aided by various species of non-crustaceans which may serve as vectors or reservoirs of WSSV [26]. These include piscivorous birds [27], molluscs [28], oysters [29], polychaetes [30,31,32], phytoplankton [33], and marine microalgae [34]. Of concern to the larger agricultural industry, piscivorous birds—due to their wide-ranging migratory and foraging routes—may serve as a vector for viruses for further ecological dispersal [35,36,37,38]. The results of a previous study suggested that feeding of domestic chickens (*Gallus gallus domesticus*) and captive seagulls (*Larus atricilla*) with WSSV-infected shrimp carcasses resulted in faecal shedding of WSSV, detected by PCR in chicken and seagull faeces for up to 57 and 72 h, respectively [27]. Currently there is no report of WSSV natural infection in domestic chickens, which is apparently due to limited surveillance of WSSV in non-crustacean hosts.

The foraging movements of gulls in South Africa [39], combined with the migration of several aquatic piscivorous birds for overwintering [40,41,42,43], pose a significant risk to disseminate exotic virus pathogens to domestic poultry, through shared roosting and foraging grounds in South Africa [44]. In this context, the present study performed metagenomic sequencing of pooled cloacal swab samples collected from domestic chickens in Makhanda, Eastern Cape, South Africa, with the aim of determining their faecal virome.

## 2. Materials and Methods

### 2.1. Ethics Approval

The study has approval from the Rhodes University Animal Research Ethics Committee; reference: 2022-5627-7014. In addition, the study has been approved by the South African Department of Agriculture, Land Reform and Rural Development (DALRRD); reference: 12/11/1/6/2/MG (2578).

### 2.2. Sample Collection

The cloacal swabs (*n* = 35) were collected in December 2022 from domestic chickens (*Gallus gallus domesticus*) in Makhanda, Eastern Cape, South Africa. Swabs were collected using sterile polyester-tipped applicators (CLASSIQSwabs™, Copan, Italy) into 1.2 mL of virus transport medium (Gibco™, Grand Island, NE, USA) in sterile 2 mL cryovials. Samples were transported on frozen ice packs in a cooler box to the laboratory and stored at −80 °C until processed.

### 2.3. Nucleic Acid Extraction

The research reported in this study was part of an overall objective of metagenomic sequencing to determine the virome of the chicken cloacal swab specimens under study. The QIAamp viral RNA mini kit (Catalogue # 52906; Qiagen, Hilden, Germany) was used for nucleic acid extraction, as Kellner et al. 2017 reported that QIAamp viral RNA mini kit was efficient for extracting both RNA and DNA fractions simultaneously, and was effective for PCR-based detection of both RNA and DNA viruses and subsequent metagenomic sequencing of extracted virus genomes [45].

In a previous study, Ladman et al. 2012 compared the pooling of five and eleven chicken swabs for real-time RT-PCR based detection of avian influenza and reported that pools of five swab specimens had consistently lower mean cycle threshold (Ct) values (higher genome copies) on a given sample day up to 14 days post-inoculation (dpi) in broiler chickens [46]. This prompted us to pool five cloacal swabs together for nucleic acid extraction. For pooling, swabs previously preserved in virus transport medium were thawed on ice, and vigorously vortexed for 1 min, followed by the pooling of 200 µL of each of five cloacal swabs into a fresh, sterile, 2 mL cryovial. A 140 µL aliquot of the pooled samples were used for nucleic acid extraction, following the manufacturer’s instructions, and eluting the extracted nucleic acids into 60 µL of nuclease-free water. Each extracted nucleic acid sample had RNA and DNA concentrations quantified using 1 µL extracted sample each on a Qubit 4 Fluorometer and Nanodrop 2000 Spectrophotometer (ThermoFisher Scientific, Waltham, MA, USA), and the remainder ~57 µL was stored at −80 °C for downstream processing.

### 2.4. Metagenomic Sequencing

The primary objective of the present study was to determine the RNA virome using domestic chicken cloacal swabs, given the abundance and severity of RNA virus diseases in domestic chickens; a protocol of virus discovery reported by Wang et al. 2003 was adopted for virus metagenomics [47], with slight modifications detailed here. In addition, our choice of nucleic acid extraction method, using the QIAamp viral RNA mini kit, provided the added advantage of sequencing DNA virus genomes simultaneously, given the efficiency of this method to extract RNA and DNA fractions simultaneously [45]. The experimental protocol consisted of three steps of enzymatic reactions. In Round A, reverse transcriptase was used for two cycles of first-strand cDNA synthesis with degenerate primer A (5′-GTT TCC CAG TCA CGA TAN NNN NNN NN-3′) procured from the Integrated DNA Technologies (Coralville, IA, USA). Sequenase version 2.0 DNA polymerase (Catalogue# 70775Z1000UN; Applied Biosystems, Waltham, MA, USA) was used for the second-strand synthesis.

During Round B, primer B (5′-GTT TCC CAG TCA CGA TA-3′), sourced from Integrated DNA Technologies (Coralville, IA, USA), was used to amplify the templates generated in Round A. In Round C, additional PCR cycles were conducted for enrichment. The details of these steps, Round A–Round C, are described in the following subsections:

#### 2.4.1. Round A—First-Strand Synthesis with Reverse Transcriptase (RT)

For reverse transcription, a 10 µL volume of 2× enzyme mix was prepared by mixing the following reagents: 2.0 µL of 10× RT buffer, 0.4 µL of 25 mM dNTP mix, 3.6 µL of nuclease-free water, 2.0 µL of 0.1 M DTT, and 2.0 µL of reverse transcriptase. All reagents for this reaction were sourced from ThermoFisher Scientific, Johannesburg, South Africa.

Into a 500 µL tube, 9 µL of extracted nucleic acid sample was added to 1 µL of primer A (40 µM, stock concentration) to a final volume of 10 µL. 10 µL of the 2× enzyme mix was added to this mixture to a final reaction volume of 20 µL. The reaction was heated to 65 °C for 5 min in a water bath, followed by cooling at room temperature for 5 min. Using a water bath, the reaction was incubated at 42 °C for 30 min, heated to 65 °C for 5 min, and then cooled at room temperature for 5 min. A further 1 µL aliquot of reverse transcriptase was added to the mixture and the reaction was placed into a thermocycler. There, it was incubated at 42 °C for 30 min, heated to 94 °C for 2 min, rapidly cooled to 10 °C, and held at 10 °C for 5 min. The reaction mixture was collected by briefly spinning it in a microcentrifuge for 5 s and then decanted into a 0.2 mL PCR tube for second-strand synthesis.

#### 2.4.2. Second-Strand Synthesis with Sequenase Version 2.0 DNA Polymerase

In a fresh 0.2 mL PCR tube, 10 µL Sequenase mix was prepared. This consisted of 2.0 µL of 5× Sequenase buffer, 7.7 µL of nuclease-free water, and 3.9 units (0.3 µL of 13 units/µL) of Sequenase enzyme (Catalogue # 70775Z1000UN; Applied Biosystems, Waltham, MA, USA). The 10 µL Sequenase mix was added to the reaction mixture from the first-strand cDNA synthesis to bring the total reaction volume to 31 µL. In a thermocycler, this reaction mixture was processed using the following program: A gradual increase in temperature from 10 °C to 37 °C over 8 min, hold at 37 °C for 8 min; a rapid ramp in temperature to 94 °C and holding at that temperature for 2 min; and finally, a rapid decrease to 10 °C and hold for 5 min at 10°C. During this hold, a further 3.9 units of Sequenase (diluted 1:4 in Sequenase buffer) was added; the reaction mixture was subsequently slowly ramped from 10 °C to 37 °C over 8 min; held at 37 °C for 8 min; followed by a ramp to 94°C and holding there for 8 min. This completed the second-strand synthesis, creating the Round A pool of samples.

#### 2.4.3. Round B—Random PCR Amplification

Round B PCR amplification was conducted using AmpliTaq Gold™ DNA polymerase with buffer II and MgCl_2_ (Catalogue # N8080247; Applied Biosystems, Waltham, MA, USA). In a fresh 0.2 mL PCR tube, the following components were mixed: Round A template (6.0 µL), 50 mM MgCl_2_ (4.0 µL), 10× PCR buffer (10.0 µL), 25 mM dNTP (1.0 µL), 40 µM primer B stock (1.0 µL), Taq polymerase (1.0 µL), and nuclease-free water (77.0 µL). The PCR program consisted of 40 cycles of 94 °C for 30 s, 40 °C for 30 s, 50 °C for 30 s, and 72 °C for 1 min. A control sample consisting of nuclease-free water (Catalogue # 129115; Qiagen, Hilden, Germany) as the template was also included.

To verify the random amplification, 9 µL of PCR products from Round B were loaded on a 1% (*w*/*v*) agarose (Catalogue # 50004; SeaKem^®^ LE Agarose, Lonza, Rockland, ME, USA) gel, prepared using 1× TAE buffer (pH 7.0), and stained with 10 µL of 10,000× GelRed (Catalogue # SCT123; Merck, Modderfontein, South Africa). The samples were mixed with 1 µL of 10× loading dye, loaded into the wells of the gel and electrophoresed at 80 V for 45 min and visualized.

#### 2.4.4. Round C—PCR Enrichment and Clean Up

A 10 µL aliquot of the Round B template was enriched via Round C, using AmpliTaq Gold™ DNA polymerase with Buffer II and MgCl_2_ (Catalogue # N8080247; Applied Biosystems, Waltham, MA, USA). In a fresh 0.2 mL PCR tube, the following components were added: Round B template (10.0 µL), 50 mM MgCl_2_ (4.0 µL), 10× PCR buffer (10.0 µL), 25 mM dNTP mix (1.0 µL), 40 µM primer B stock (1.0 µL), Taq DNA polymerase (1.0 µL), and nuclease-free water (73.0 µL) to a final volume of 100 µL. The PCR program consisted of 20 cycles of 94 °C for 30 s, 40 °C for 30 s, 50 °C for 30 s, and 72 °C for 1 min.

PCR products from Round C were separated using the same electrophoresis conditions detailed in Section 2.4.3. Following visualization under transillumination, the band of the enriched DNA corresponding between 0.5 and 1.0 kb was manually excised and purified using the Wizard^®^ SV Gel and PCR Clean-Up System (Catalogue # A9281; Promega, Madison, WI, USA). The gel-purified DNA was quantified using Qubit 4 Fluorometer (ThermoFisher Scientific, Waltham, MA, USA) and Nanodrop 2000 Spectrophotometer, and was used for library preparation for Nanopore MinION sequencing.

#### 2.4.5. Nanopore MinION Library Preparation and Sequencing

The gel-purified DNA were used for Nanopore MinION library preparation using SQK-NBD114.96 library preparation kit, barcoded for multiplexing, and sequenced on a FLOMIN114 flow cell (R10.4.1) in a MinION Mk 1B instrument, connected to a desktop computer, following the manufacturer’s instructions.

#### 2.4.6. Genome Assembly and Annotation

The .FASTQ files containing Nanopore single-end reads were analysed using Genome Detective - panviral 2.52 [48]. Briefly, Genome Detective performed quality control, filtered out non-viral reads, and conducted de novo assembly of viral reads into groups representing the lowest common ancestor using NCBI Taxonomy. The Genome Detective aligned the resulting contigs to the NCBI reference genomes using the codon-aware pairwise alignment tool – Advanced Genome Aligner (AGA) [49] and calculated the consensus sequences. The annotation of assembled WSSV genome was conducted in ‘Geneious Prime 2023.1.2’ software (Biomatters, Auckland, New Zealand) using reference genome (GenBank accession: NC_003225.3) and Genome Annotation Transfer Utility (GATU) [50]. Percent protein identity between South African WSSV and other WSSV genomes included in this study (*n* = 29) was determined using GATU.

### 2.5. Phylogenetic Analysis

This study reports only on the WSSV genome from the above assembled genomic information. The assembled WSSV genome was manually analysed in ‘Geneious Prime 2023.1.2’ software to determine percent nucleotide identity, insertions, and deletions compared to the reference genome. Complete WSSV genome sequences (*n* = 29) available on the NCBI-GenBank were downloaded and aligned with the South African WSSV genome using the MUSCLE algorithm in Molecular Evolutionary Genetics Analysis (MEGA) version X software [51] to find the best model for the phylogenetic analysis. The Hasegawa–Kishino–Yano (HKY) model was used to construct the Neighbour-Joining tree, with 1000 bootstrap replications, in ‘Geneious Prime 2023.1.2’.

### 2.6. Principal Coordinates Analysis (PCoA)

The distance matrix for the nucleotide sequence alignment of WSSV genomes (*n* = 30) was generated using the MUSCLE algorithm in ‘Geneious Prime 2023.1.2’ software. The distance matrix generated a comma-separated values (CSV) file and was converted to an Excel file using Microsoft Excel. This was used for the principal coordinates analysis (PCoA) using covariance in ‘PAST: Paleontological Statistics Software Package for Education and Data Analysis’ version 4.02 software [16].

## 3. Results

The cloacal swab specimens were collected from 8-week-old broilers and layers under the supervision of a State Veterinarian. During sampling, most broilers appeared healthy, and only a few of them (*n* = 4) appeared lethargic. The layers were free-range and largely appeared healthy, except for a few layer chickens (*n* = 6) that had been segregated from other free-range chickens and confined to a separate area. This group’s symptoms included lethargy, exhibiting paleness, reduced appetite, and ruffled feathers. The cloacal swabs were collected from all the apparently sick, or lethargic chickens along with randomly selected apparently healthy chickens.

The Qubit 4 Fluorometer quantification after extraction measured concentrations of RNA ranging between 4.76 and 10.40 ng/µL (average ± standard deviation = 7.32 ± 2.31 ng/µL), while the DNA content ranged between 5.17 and 11.32 ng/µL (8.50 ± 2.24 ng/µL). To determine the diversity of viruses in chicken cloacal swab specimens under study, the cloacal swab specimens were pooled (*n* = 7 pools) before nucleic acid extraction in the laboratory, each pool consisting of five individually collected cloacal swabs originated from 35 apparently healthy or sick chickens (i.e., exhibiting clinical signs of illness) for metagenomic sequencing, using Nanopore MinION following a SISPA protocol. While all the pools generated several genomes of animal viruses, this study reports a WSSV genome generated from one cloacal swab pool collected from apparently healthy, free-range chickens.

Briefly, we used a previously reported SISPA protocol [47], for simultaneous generation of RNA (using cDNA) and DNA virus genomes in a single assay, with the objective of developing a scalable method for studying the faecal virome of domestic chickens. The study protocol consisted of three steps, including extraction of RNA and DNA from the samples, first- and second-strand synthesis of cDNA from RNA molecules in the sample, followed by random PCR amplification of the cDNA and extracted DNA and enrichment of DNA. A previous study by Kellner et al. 2017 [45]—comparing three nucleic acid extraction methods for simultaneous detection of RNA and DNA virus genomes via metagenomic sequencing—reported that this approach (particularly, nucleic acid extraction using QIAamp’s viral RNA mini kit) detected RNA and DNA virus genomes simultaneously.

**Figure 1:**
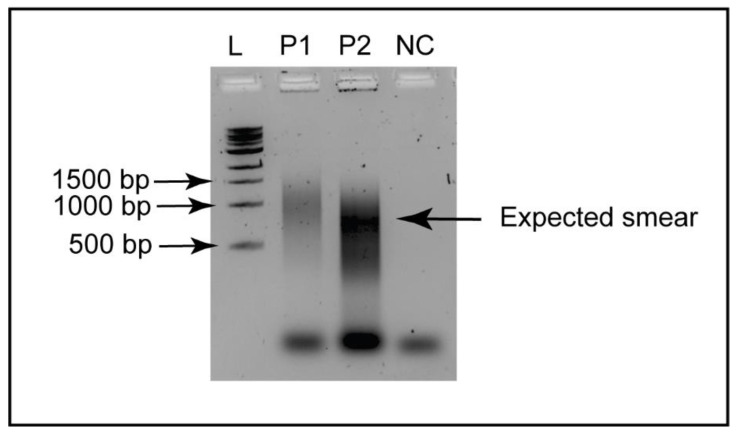
A representative image of agarose gel electrophoresis depicting the results of Round B random PCR amplification, for two pooled chicken cloacal swab specimens. Presence of a smear between approximately 500 bp to 1000 bp was considered as an indication of positive random PCR amplification, using primer B, following a SISPA protocol. (L = 1 kb DNA ladder, P1 = pooled sample 1 collected from apparently healthy chickens, P2 = pooled sample 2 collected from sick chickens, NC = negative control template).

The samples following SISPA were quantified using a Qubit 4 Fluorometer and/or agarose-gel electrophoresis. Figure 1 depicts a representative image of Round B random PCR amplification which generated a DNA smear between approximately 500 bp and 1000 bp, taken as an indicative of positive random PCR amplification.

The randomly amplified DNA was enriched using 20 additional PCR cycles before gel purification of DNA. The gel-purified DNA samples were used for Nanopore MinION library preparation using the SQK-NBD114.96 library preparation kit and barcoded for multiplexing. The sequencing was performed for 72 h on a MinION Mk1B device, generating a total of approximately 8 GB .FASTQ files, for seven pooled chicken cloacal swab specimens. While genome sequence analyses determined near full-length or partial genomes of several animal viruses, the present paper reports on a single WSSV genome, sequenced from a pooled cloacal swab specimen which generated a 1.05 GB .FASTQ file comprising 766,791 raw reads with read lengths ranging between 70 and 86,932 bp. A total of 7619 (1%) of the raw reads were directly assigned to candidate reference genomes for viral genome assembly, while 86,487 (11.28%) reads were aligned when assembled into contigs. Alignment generated several partial animal virus genomes, including the WSSV genome that is the focus of this report.

**Figure 2:**
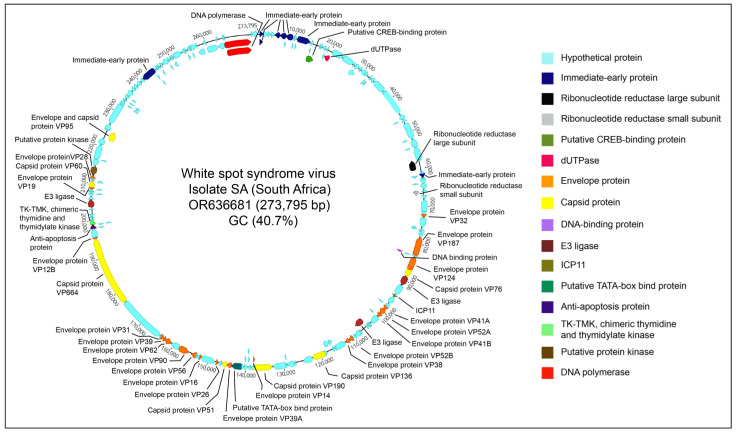
A schematic representation of South African WSSV genome (GenBank accession: OR636681; 273,795 bp) generated from a cloacal swab pool of domestic chickens. The map was generated using Genome Annotation Transfer Utility (GATU) [50] and Geneious Prime.

The generated South African WSSV genome was 273,795 bp in size (GenBank accession: OR636681), consisting of 158 open reading frames (ORFs). The genome sequence alignment using 29 complete WSSV genomes available at NCBI-GenBank suggested a 99.94% nucleotide similarity—with 88.5% genome coverage—of this South African WSSV genome with the WSSV reference genome, reported in 2018 from China (GenBank accession: NC_003225.3). This is of significance given that there is no report of the occurrence of WSSV in domestic chickens. A schematic representation of South African WSSV genome generated in this study is provided in Figure 2.

In accordance with the high degree of nucleotide consensus, the South African isolate of WSSV shared 100% amino acid identity for all proteins, including capsid proteins, envelope proteins, and DNA polymerase with the reference genome reported in 2018 from China (GenBank accession: NC_003225.3); however, there were variations in identities with certain proteins of other WSSV isolates reported on the NCBI-GenBank. More information on the amino acid identities for certain key proteins between South African WSSV genome (GenBank accession: OR636681) and selected other WSSV genomes is provided in Table 1. While most proteins, among Asian isolates, shared high similarities, certain proteins were slightly variable, such as capsid protein VP76 (87,052–89,076), capsid protein VP136 (118,623–122,282), capsid protein VP190 (133,055–137,752), capsid protein VP664 (177,046–195,279), and immediate-early protein (240,901–244,632). An Indian isolate of WSSV (GenBank accession: MG702567.1) had low amino acid identities in most proteins; with envelope protein (207,820–208,299) having the lowest similarity (60.4%). Of all the WSSV isolates analysed, the Indian isolate had the greatest difference in amino acid composition of key proteins, when compared with the South African WSSV isolate. The following Table 1 represents amino acid identities in certain key proteins for selected WSSV isolates.

The comparison of amino acid identities of key proteins between the South African and other WSSV genomes (Table 1) suggested that some variation in encoded amino acids occurred between the South African genome and those found in differing geographical regions, except for the WSSV genome reported from China (GenBank accession: NC_003225.3). This further supports the close genetic proximity between the South African WSSV genome reported in this study with the reference genome reported from China.

While there were variations in amino acid identities in most key proteins, DNA polymerase appeared to be quite conserved, with 100% amino acid identity, for most WSSV isolates, only with slight variations for two isolates reported from China (GenBank accession: KY827813.1) and Japan (GenBank accession: AP02728.1) where amino acid identities for the DNA polymerase gene with South African WSSV isolate were 99.9%. To note, two WSSV isolates, reported from Australia (GenBank accession: MF768985.1) and India (GenBank accession: MG702567.1) had significant variations in DNA polymerase gene compared to all other WSSV isolates, including the South African isolate generated in this study. While all WSSV genomes had one DNA polymerase gene, the reported Indian isolate had multiple ORFs for DNA polymerase, most of which were suggested to be nonfunctional DNA polymerase due to mutations, described in the GenBank annotation. In addition, the Australian and Indian WSSV isolates also had significant variations in other proteins compared to all other WSSV isolates reported on NCBI-GenBank.

**Figure 3:**
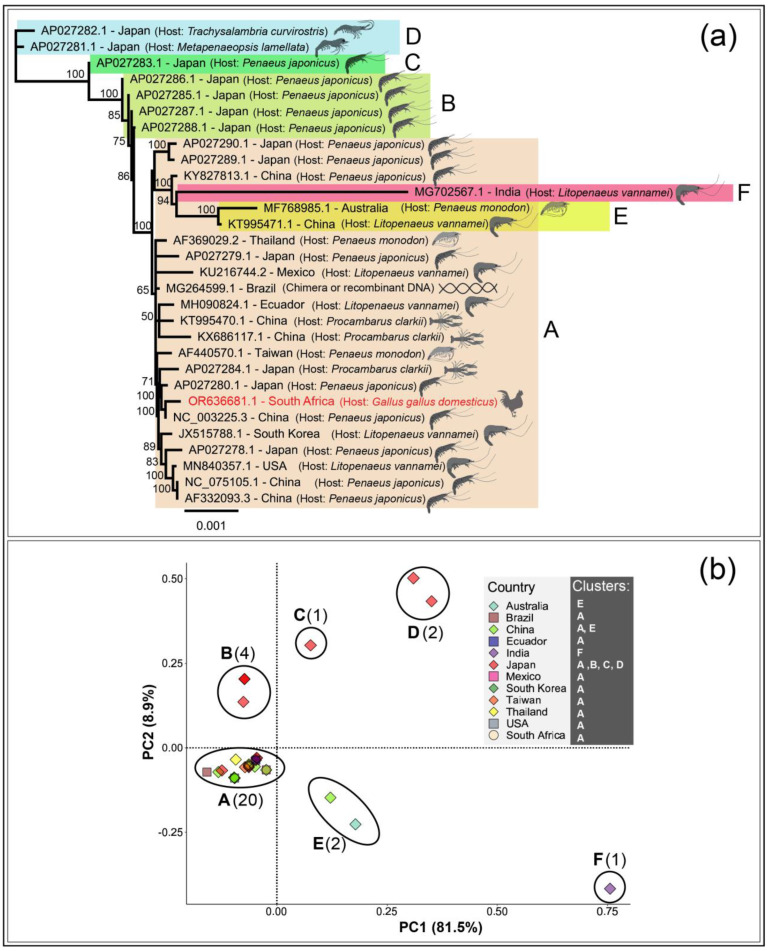
(**a**). The Neighbour-Joining tree of full-length WSSV genome sequences reported on NCBI-GenBank (*n* = 29) and the South African WSSV genome (GenBank accession: OR636681) generated in this study. The phylogeny suggested multiple origins of WSSV genomes and grouped the WSSV genomes into six clusters (A–F). The South African WSSV genome grouped with WSSV genomes reported from South East Asia, including China and Japan. (**b**). Principal coordinate analysis (PCoA) suggested the occurrence of WSSV genomes, reported globally, in six clusters (A–F), supporting the phylogenetic branching of WSSV genomes. In PCoA, most WSSV genomes (*n* = 20), including the South African WSSV genome, aligned in cluster A. The South African WSSV genome overlapped with the WSSV genomes reported from South East Asian countries, including China and Japan. Clusters B, C, and D grouped WSSV genomes reported from Japan only. Cluster E grouped WSSV genomes reported from China and Australia. Cluster F had a single WSSV genome reported from India.

The full-length WSSV genomes deposited at NCBI-GenBank in previous studies were isolated from several crustacean hosts in various countries. For instance, the WSSV genomes were generated from the kuruma shrimp or Japanese tiger prawn (*Penaeus japonicus*, also known as *Marsupenaeus japonicus*), reported from Japan and China. The WSSV genomes were also generated from giant tiger prawn (*Penaeus monodon*), reported from Australia, Taiwan, and Thailand. Other WSSV genomes were generated from the Whiteleg shrimp also known as King prawn (*Litopenaeus vannamei*), reported from China, Ecuador, India, Mexico, South Korea, and the USA. The WSSV genomes were also generated from red swamp crayfish (*Procambarus clarkii*), reported from China and Japan. Two more WSSV genomes reported from Japan were generated from a shrimp (*Trachysalambria curvirostris*) and a velvet shrimp (*Metapenaeopsis lamellata*). To note, most of the full-length WSSV genomes available on NCBI-GenBank were reported from Japan (13/29), generated from four species of shrimps. The Neighbour-Joining tree grouped the South African WSSV genome with the WSSV genomes reported from *Penaeus japonicus* in China and Japan (Figure 3a). Precisely, the South African WSSV genome clustered with the WSSV genome reported from a shrimp (*Penaeus japonicus*) in China in 2018 (GenBank accession: NC_003225.3). Largely, the phylogenetic tree grouped the South African WSSV genome with the WSSV genomes reported from South East Asian countries. In addition, the phylogenetic tree observed multiple clusters of WSSV genomes reported worldwide in various hosts.

The phylogenetic clustering of South African WSSV isolate (GenBank accession: OR636681) with WSSV isolate reported from China (GenBank accession: NC_003225.3) was also supported by the protein identity across the 158 ORFs of the South African WSSV genome, with results presented in Table 1 for certain key proteins. In addition, the divergence of the Indian isolate (node F; GenBank accession: MG702567.1) and Australian isolate (node E; GenBank accession: MF768985.1) as observed in the phylogenetic tree (Figure 3a) could be explained by the occurrence of significant variations, such as insertions and deletions of ORFs, and lower protein identities of these two genomes compared to other WSSV genomes.

The principal coordinate analysis (PCoA) suggested at least six clusters (A–F) of reported full-length WSSV genomes available at the NCBI-GenBank (Figure 3b). Most of the WSSV genomes (20/30, i.e., 66.67% of available genomes), including the South African WSSV genome reported in this study, were grouped in cluster A. WSSV genomes within this cluster spanned multiple global regions (South East Asia, the Americas and Africa) and were isolated from a wide variety of crustacean hosts, e.g., *P*. *japonicus, P. clarkii, L. vannamei*, etc., as well as *the G. gallus domesticus*, non-crustacean host reported here. Particularly, the South African WSSV genome overlapped closely with the other WSSV genomes reported from South East Asian countries, such as China, Japan, and Taiwan. This observation supported the phylogenetic grouping of the South African WSSV genome with WSSV genomes reported from South East Asian countries.

The PCoA plot grouped several WSSV genomes reported from Japan into four clusters, viz., clusters A, B, C, and D. For WSSV identified in Japan, component location along the PCoA plot appeared to depend strongly on host: while samples in cluster A (AP027280.1 and AP027279.1) were isolated from both *P. japonicus* (prawns) and *P. clarkii* (lobster), the closely related samples in cluster B and the single sample forming cluster C were isolated from only *P. japonicus*, while Cluster D’s members were isolated from other crustaceans. The distribution of these clusters, and the variety of hosts from which these WSSV genomes were isolated from, may indicate a large and diverse reservoir of this virus occurring naturally in this region, or may be the result of targeted studies of WSSV by this country, due to the agricultural and economic importance of crustaceans to this region.

Scattered clusters that showed genetic divergence from the others included E (samples originating from Australia and China and infecting diverse crustacea) and F (a single sample isolated from India infecting *L. vannamei* hosts). Here, it was of interest to note that the WSSV genomes reported from Japan (*n* = 13) grouped into four separate clusters, some of which overlapped in cluster A, suggesting the possibility of host-specific adaptation and variations among WSSV genomes. Overall, the PCoA plot suggested multiple origins of WSSV genomes reported worldwide. Also, the grouping of various WSSV genomes generated from different geographical locations globally, in cluster A, again suggested the potential of host-specific adaptation of WSSV.

In summary, the present study generated a 273,795 bp genome of WSSV from a cloacal swab pool of free-range domestic chickens in Makhanda, Eastern Cape, South Africa, using Nanopore MinION sequencing. The South African WSSV genome has close nucleotide similarity (99.94%) with a WSSV genome reported from China in 2018 and clustered with WSSV genomes reported from South East Asia. To our knowledge, this is the first study that generated a WSSV genome from domestic chickens.

## 4. Discussion

The white spot disease (WSD) caused by WSSV causes serious economic losses to the shrimp-farming industry globally [2,52]; WSD outbreaks occur in crustaceans worldwide, including countries in Asia [1,12,13,14], Africa, the Middle-East [53,54], Europe [16], Australia [17], North America [55,56], Central America [20], and South America [18,19]. The available literature suggests that while crustacean hosts may serve as the primary reservoir of WSSV, several non-crustacean species, such as piscivorous birds [27], molluscs [57], and aquatic insects [30,31,32] may serve as the carrier of WSSV and therefore may facilitate further ecological dissemination. The present metagenomic sequencing study generated a WSSV genome from a pool of cloacal swab samples of domestic chickens under study. Of note, WSSV infects crustaceans, including shrimps [2,3,4,12,58,59], prawns [17,58], crabs [15,60], crayfish [21], and lobsters [22,23,61]. A previous experimental study reported feed-related transmission of WSSV to domestic chickens, after experimental feeding upon WSSV-infected shrimp carcasses, and detection of WSSV DNA in chicken faeces up to 57 h after feeding [27]. The current study is the first report of the occurrence of WSSV in a cloacal swab pool of domestic chickens. This raises the question of the diet of the free-range domestic chickens under study. The farmer noted that the domestic chickens were fed commercial poultry feed (sourced from Rayner General Agencies Cc, Makhanda, Eastern Cape, South Africa). According to the manufacturer, broiler feed and laying mash were prepared in-house, using ingredients sourced from within South Africa, stating furthermore that their products did not contain animal-derived ingredients. A “poultry mix” feed supplied through the company contained a mixture of various seeds and grains, including sunflower seeds, sorghum, millet, and corn. The farmer indicated that the chickens were also occasionally fed other plant-derived produce including cabbage and spinach leaves. This diet minimises the possibility of feed-related transmission of WSSV to the chickens.

Freshwater crustaceans, the marron crayfish (*Cherax chanini*), are farmed commercially in the Eastern Cape, at a site over 150 km from Makhanda. However, to our knowledge, no currently active commercial marine crustacean farming occurs in the region of the present study. Just under twenty years ago, a commercial shrimp-farming operation closed down in the province of KwaZulu Natal, situated over 500 km from the study site. Species of shrimp (*Penaeus japonicus* and *Penaeus indicus*) and oysters (*Crassostrea gigas*) were reported to be farmed in KwaZulu-Natal [62]. In addition, in Southern Africa, Mozambique [54] and Madagascar [53,54] are two prominent shrimp-producing countries, with geographical proximity to South Africa. Of particular note was a commercial shrimp farm situated in Coega, approximately 110 km from the study site. Closed in 2009, this facility farmed the Pacific white shrimp (*Litopenaeus vannamei*).

Previous studies reported genotypic diversity of WSSV in Madagascar and Mozambique [53,54]; however, these studies only reported partial WSSV gene sequences and therefore were not included in the present study, since we utilized full-length WSSV genome sequences in this study. It is noteworthy that intercontinental migration of wild and aquatic birds has been linked to virus dispersal and spillover to domestic avian species, including domestic chickens [63,64,65,66,67,68]. Previous studies have reported various flyways through which several avian species migrate to overwinter in different parts of the world [69,70,71,72,73]. Several hotspots for avian virus spillover occur in Africa on migratory bird flyways [74,75,76,77,78,79,80]. Since numerous species of wild and aquatic birds overwinter each year in South Africa, this poses a serious threat of introduction and spillover of exotic virus pathogens to domestic avian species within South African territory [81]. The overwintering of migratory wild birds in Southern Africa is facilitated by different migration routes, including the African–Eurasian flyway [75], East-Atlantic flyway [82], and East Africa–West Asia flyway [83]. One possible explanation of the close similarity between the South African WSSV isolate with the Chinese strain CN01 of WSSV might be due to the spillover from migratory birds, serving as a vector, through shared foraging and roosting grounds with free-range chickens.

Since most sequences of WSSV reported on the NCBI-GenBank are predominantly from marine crustacea, it is noted that the specific sampling site is situated near Makhanda which is around 50 km from coastal areas. While quite unusual, waders that forage for crustaceans (including shrimp) along shorelines, such as plovers and sandpipers have been noted in areas around Makhanda where small bodies of water are present. However, at this stage, the theory of possible avian transmission can only be hypothesized; this requires further investigation, such as PCR-based detection of WSSV in bird droppings from the study site. Another limitation of the present study is the lack of data on molecular detection of WSSV in chicken cloacal swab specimens. This is because the present study was primarily aimed at detecting important RNA virus pathogens, known to cause devastating damage to chicken farming worldwide, including South Africa. Therefore, we adopted a previously reported SISPA protocol for virus discovery [47] with the primary aim of exploring the diversity of RNA viruses in chicken samples under study. The sequencing of a DNA virus genome is an expected outcome given that previous reports using the same extraction [45] and similar amplification protocols, using SISPA, also reported sequencing a combination of RNA and DNA viral genomes [84].

Since we detected the WSSV genome in a pool of cloacal swab specimens of chickens, this raises the question of whether WSSV may have a faecal–oral circulation route amongst avian species, similar to several other avian viruses such as avian influenza. Answering this question would require further studies through experimental infection of chickens. The phylogenetic comparison of full-length WSSV genomes available at the NCBI-GenBank up to September 2023 suggests a generally high genetic variability among WSSV strains, which might be influenced more strongly by host-specific adaptations than by geographic restrictions, reinforcing the possibility that these strains might be widely and rapidly transmitted between these countries, such as migratory birds.

There are 29 complete genome sequences of WSSV available in the NCBI-GenBank database until September 2023. The WSSV genome size varies between strains, ranging from approximately 281 kb, reported in 2018 from India (NCBI-GenBank accession: MG702567) [85], to 314 kb, reported in 2015 from Brazil (MG264599) [86]. A similar variation occurs in the number of protein-coding genes or open reading frames (ORFs) between strains: for example, Yang et al. 2001 reported a complete WSSV genome of approximately 305 kb that encompassed 181 ORFs [1]; van Hulten et al. 2001 provided a complete WSSV genome of approximately 293 kb that encompassed 184 ORFs (AF369029) [87]; Li et al. 2017 reported three complete WSSV genomes comprising 177, 164, and 154 ORFs, all three of these varying in virulence [88]. The South African WSSV genome of 273,795 bp encodes 158 protein-coding genes, within range of the previous reports, given the diversity of this virus.

To our knowledge, one study utilized WSSV-infected shrimp carcasses for feeding domestic chickens and captive seagulls (*Larus atricilla*), which resulted in faecal shedding of WSSV, detected by PCR in chicken and seagull faeces for up to 57 and 72 h, respectively [27]. Investigating the pathogenesis and virulence of WSSV in domestic chickens would require further studies, such as necropsy of experimentally infected chickens that experience viral shedding and/or exhibit clinical signs of illness. This could be used for determining systemic infection of WSSV in chickens, along with examining the presence of macroscopic or microscopic lesions in internal organs of chickens, if any. Histopathological investigations would be useful to ascertain tissue tropism of WSSV infection in domestic chickens. The present finding of the WSSV genome in domestic chickens opens a new avenue of research for understanding the pathogenesis of WSSV in chickens and whether it can replicate and cause clinical disease in domestic chickens.

Future studies may investigate potential routes of WSSV inter-species transmission. Virus surveillance in migratory piscivorous wild birds may explore their role in the dissemination of exotic virus pathogens in Southern Africa, and related implications for domestic avian species. The significance of WSSV transmission to domestic chickens and its ability to cause systemic infection and replication would require further investigations. The present study had certain limitations: the surveillance was conducted in a small geographical area; and poultry flocks were sampled only once and not throughout the year. Active surveillance of domestic chickens, using SISPA based metagenomic sequencing, would be beneficial for understanding the persistence and circulation of virus pathogens.

## 5. Conclusions

To our knowledge, for the first time, this study reports a genome of WSSV in domestic chickens (*Gallus gallus domesticus*) sampled in the Eastern Cape, South Africa. This suggests the possibility of expansion of the WSSV host range. Further experimental studies would determine the pathogenesis and significance of WSSV infection in domestic chickens. Future studies should consider WSSV genotyping which may be useful for determining transmission dynamics of WSSV in domestic chickens. It will also be interesting to conduct a molecular surveillance for virus detection in migratory wild birds in the region, to explore the prevalence of WSSV infection and to validate the risk of WSSV dissemination associated with avian migration.

## Figures and Tables

**Table 1:** Percent amino acid identities of key proteins encoded by South African WSSV isolate (GenBank accession: OR636681) and WSSV genomes reported from different geographical regions.

WSSV–SA (GenBank Accession: OR636681)			Frame	Percent Protein Identity: GenBank Accession (Country)						
Protein	Start (bp)	End (bp)		NC_003225.3 (China)	AP027290.1 (Japan)	AF369029.2 (Thailand)	AF440570.1 (Taiwan)	MF768985.1 (Australia)	KU216744.2 (Mexico)	MN840357.1 (USA)
Immediate-early protein	629	1303	Forward	100.0	99.6	100.0	100.0	100.0	100.0	100.0
dUTPase	18,927	20,312	Reverse	100.0	100.0	100.0	100.0	98.7	100.0	100.0
Envelope protein VP53B	20,510	23,416	Forward	100.0	100.0	99.9	100.0	100.0	100.0	100.0
Ribonucleotide reductase large subunit	57,563	60,109	Reverse	100.0	100.0	100.0	100.0	100.0	100.0	100.0
Ribonucleotide reductase small subunit	65,794	67,035	Forward	100.0	100.0	100.0	100.0	100.0	99.8	100.0
Envelope protein VP32	72,090	72,926	Forward	100.0	100.0	100.0	100.0	100.0	100.0	100.0
Envelope protein VP187	78,128	82,948	Reverse	100.0	100.0	100.0	100.0	99.8	99.9	100.0
DNA-binding protein VP15	83,105	83,290	Forward	100.0	100.0	100.0	100.0	90.9	100.0	100.0
Envelope protein VP124	83,398	86,982	Reverse	100.0	99.9	100.0	99.9	99.2	99.9	100.0
Capsid protein VP76	87,052	89,076	Forward	100.0	100.0	99.9	99.9	98.0	100.0	100.0
E3 ligase	89,095	91,629	Forward	100.0	100.0	100.0	100.0	99.9	99.8	100.0
Envelope protein VP41A	98,576	99,454	Forward	100.0	100.0	100.0	100.0	NE*	100.0	100.0
Envelope protein VP52A	99,491	100,951	Forward	100.0	100.0	98.8	100.0	NE*	100.0	100.0
Envelope protein VP41B	101,003	101,905	Forward	100.0	100.0	100.0	100.0	NE*	100.0	100.0
Envelope protein VP52B	110,480	111,634	Forward	100.0	100.0	100.0	100.0	100.0	99.7	100.0
Envelope protein VP38	111,695	112,624	Forward	100.0	100.0	100.0	100.0	100.0	100.0	100.0
Capsid protein VP136	118,623	122,282	Forward	100.0	99.9	99.9	100.0	99.8	99.8	100.0
Capsid protein VP190	133,055	137,752	Reverse	100.0	99.9	99.9	98.7	99.4	99.8	100.0
Envelope protein VP14	137,706	137,999	Reverse	100.0	100.0	100.0	100.0	100.0	100.0	NE*
Putative TATA-box bind protein	141,117	143,792	Forward	100.0	99.9	100.0	100.0	100.0	100.0	100.0
Envelope protein VP39A	143,779	145,038	Forward	100.0	100.0	100.0	100.0	91.3	100.0	100.0
Capsid protein VP51C	145,063	146,463	Forward	100.0	100.0	99.8	100.0	100.0	100.0	100.0
Envelope protein VP26	147,361	147,975	Reverse	100.0	100.0	100.0	100.0	100.0	100.0	100.0
Envelope protein VP16	152,071	152,424	Forward	100.0	100.0	100.0	100.0	95.1	100.0	100.0
Envelope protein VP56	153,372	154,769	Forward	100.0	100.0	100.0	100.0	100.0	100.0	99.8
Envelope protein VP90	155,603	158,173	Reverse	100.0	100.0	100.0	100.0	100.0	100.0	100.0
Envelope protein VP11	160,758	162,059	Reverse	100.0	100.0	100.0	100.0	NE*	99.8	100.0
Envelope protein VP39	162,082	162,933	Reverse	100.0	100.0	100.0	100.0	NE*	100.0	100.0
Envelope protein VP31	162,937	163,722	Reverse	100.0	99.6	100.0	100.0	100.0	100.0	100.0
Capsid protein VP664	177,046	195,279	Forward	100.0	100.0	99.9	99.9	99.8	100.0	100.0
Envelope protein VP12B	195,419	195,625	Reverse	100.0	100.0	100.0	100.0	80.0	100.0	100.0
Anti-apoptosis protein	198,046	199,011	Forward	100.0	100.0	100.0	100.0	99.7	100.0	100.0
Envelope protein VP19	208,379	208,744	Reverse	100.0	100.0	99.2	100.0	100.0	100.0	100.0
Capsid protein VP60B	208,882	210,516	Forward	100.0	100.0	100.0	100.0	99.1	100.0	100.0
Envelope protein VP28	211,349	211,963	Forward	100.0	100.0	100.0	100.0	99.5	100.0	100.0
Putative protein kinase	212,058	214,250	Reverse	100.0	100.0	100.0	100.0	100.0	100.0	100.0
Envelope and capsid protein VP95	222,182	224,584	Forward	100.0	100.0	100.0	100.0	99.9	99.8	100.0
Immediate-early protein	240,901	244,632	Reverse	100.0	99.6	99.9	99.8	99.8	99.8	99.9
DNA polymerase	265,017	272,072	Forward	100.0	100.0	100.0	100.0	100.0	100.0	100.0
Structural protein VP55	272,381	273,727	Reverse	100.0	100.0	100.0	99.8	99.8	99.8	100.0

NE* = Not Encoded.

## Data Availability

The South African WSSV genome generated in this study is available at NCBI-GenBank; accession number OR636681.
